# Under-Vaccination in Pediatric Liver Transplant Candidates with Acute and Chronic Liver Disease—A Retrospective Observational Study of the European Reference Network *TransplantChild*

**DOI:** 10.3390/children8080675

**Published:** 2021-08-03

**Authors:** Tobias Laue, Zeynep Demir, Dominique Debray, Mara Cananzi, Paola Gaio, Valeria Casotti, Lorenzo D’Antiga, Vaidotas Urbonas, Ulrich Baumann

**Affiliations:** 1 Department of Paediatric Liver, Kidney and Metabolic Diseases, Division of Paediatric Gastroenterology and Hepatology, Hannover Medical School, 30625 Hannover, Germany; Baumann.U@mh-hannover.de; 2 Pediatric Hepatology Unit, Necker Enfants Malades Hospital, 75015 Paris, France; zeynep.demir-ext@aphp.fr (Z.D.); dominique.debray@aphp.fr (D.D.); 3 Unit of Paediatric Gastroenterology, Digestive Endoscopy, Hepatology and Care of the Child with Liver Transplantation, University Hospital of Padova, 35142 Padova, Italy; maracananzi@yahoo.com (M.C.); paola.gaio@aopd.veneto.it (P.G.); 4 Pediatric Hepatology, Gastroenterology and Transplantation Unit, Hospital Papa Giovanni XXIII, 24127 Bergamo, Italy; vcasotti@asst-pg23.it (V.C.); ldantiga@asst-pg23.it (L.D.); 5 Clinic of Children’s Diseases, Vilnius University Faculty of Medicine, 08406 Vilnius, Lithuania; vaidotas.urbonas@santa.lt

**Keywords:** pediatric liver transplantation, vaccination, immunization, *TransplantChild*

## Abstract

Infection is a serious concern in the short and long term after pediatric liver transplantation. Vaccination represents an easy and cheap opportunity to reduce morbidity and mortality due to vaccine-preventable infection. This retrospective, observational, multi-center study examines the immunization status in pediatric liver transplant candidates at the time of transplantation and compares it to a control group of children with acute liver disease. Findings show only 80% were vaccinated age-appropriately, defined as having received the recommended number of vaccination doses for their age prior to transplantation; for DTP-PV-Hib, less than 75% for Hepatitis B and two-thirds for pneumococcal conjugate vaccine in children with chronic liver disease. Vaccination coverage for live vaccines is better compared to the acute control group with 81% versus 62% for measles, mumps and rubella (*p* = 0.003) and 65% versus 55% for varicella (*p* = 0.171). Nevertheless, a country-specific comparison with national reference data suggests a lower vaccination coverage in children with chronic liver disease. Our study reveals an under-vaccination in this high-risk group prior to transplantation and underlines the need to improve vaccination.

## 1. Introduction

Infection is still the most common cause of mortality in the long term after liver transplantation, despite the advances in immunosuppression and medical management [1,2]. The society of pediatric liver transplantation (SPLIT) in the United States registered almost 38% culture-proven infections within the first 90 days of transplantation [3]. Moreover, of the 3.8% of patients who died, almost 12% had an infection. At Bicêtre University Hospital, 47% of children suffered from bacterial infections in the early phase after pediatric liver transplantation, leading to death in 3% of patients [4]. A recent US-multicenter study showed that 16% of all pediatric solid organ transplant recipients suffered at least one hospitalization for a vaccine-preventable infection (VPI) in the first 5 years after transplantation, resulting in increased morbidity and mortality [5]. Moreover, a prolonged hospitalization after transplant due to VPI increased costs on average of about USD 120,498. Leading VPI were influenza (7.2%), rotavirus (3.7%), varicella (2.1%) and pneumococcus (2.0%). Thus, vaccination represents an easy, less invasive and cheap opportunity to reduce infections in children before and after transplantation.

However, based on national recommendations, only 55% of US [6] and 70% of Swiss [7] patients were up to date with immunization, before orthotopic liver transplantation (OLT). The window of opportunity for vaccination is usually limited to prior to transplantation because pediatric liver transplant candidates often have unstable disease courses. Thus, it is paramount that eligible children are immunized at an early age.

This study aims to examine the immunization coverage in pediatric patients who underwent OLT at five liver transplant centers in Europe. An age-appropriate vaccination is considered, when patients have received the recommended number of vaccination doses for their age prior to transplantation. In order to investigate if children with chronic liver disease are vaccinated according to national vaccination guidelines, the vaccination rates of these children are compared to those of children with acute onset liver disease. This cohort of children serves as controls. To reduce country-specific variation, vaccination coverage is compared with national reference data. We also analyzed antibody titers before transplantation.

## 2. Materials and Methods

### 2.1. Patients and Data Acquisition

This multi-center, retrospective analysis included 430 children who were born between 1995 and 2020 and who underwent liver transplantation at University Hospital of Padova (Italy), Hospital Papa Giovanni XXIII in Bergamo (Italy), Necker Enfants Malades Hospital in Paris (France), Vilnius University Clinic of Children’s Diseases (Lithuania) and Hannover Medical School (Germany). Only children with a certified immunization record and aged below 18 years at the time of transplantation were included in this study. All parents/caregivers of patients analyzed in this study provided informed consent allowing their children’s data to be used for scientific purposes at the time of hospital admission. Patient data were anonymized prior to analysis. Ethical approval was not necessary due to the retrospective design of the study, according to European legislation.

Vaccination dates were taken from vaccination records. To assess whether the patient received an age-appropriate vaccination prior to transplantation, the national vaccination recommendations at time of birth were used. If the age limit is changed here (e.g., lowering the age for booster dose with MMR from 3 years to 16 months), the new limit applies to all who have not yet been transplanted and who exceed this age limit. With EU approval of a new vaccine (e.g., against meningococcal B or rotavirus), the age limits for a vaccination set by the manufacturer count, as long as no national vaccination recommendation is made. If the vaccine is implemented in the national vaccination calendar, these age limits are used to classify an age-appropriate vaccination status. Children were considered as age-appropriate vaccinated if they had received the recommended doses of a vaccine required for their age, as mentioned above, by the time of the liver transplantation.

Antibody titers against Hepatitis A and B, as well as measles and varicella, were determined pre-transplant. Those patients who received albumin, fresh frozen plasma or immunoglobulins before antibody measurement were excluded, as well as children under 6 months of age, due to potential maternal antibodies. Depending on levels and as specified by the manufacturer for each test, they were considered as non-immune or immune. Borderline IgG was considered as non-immune, due to the long observation period in several centers and the adjustments to the reference ranges after test changes.

### 2.2. Statistical Analysis

Qualitative data was expressed as number and percentage (%). Quantitative data was expressed as median (25–75% quartile). The comparison of two groups with categorical variables was performed using chi-squared test or Fisher’s exact test. Mann–Whitney U test was used for continuous variables due to non-normality. *p* < 0.05 was considered statistically significant. Statistical analysis was performed using R version 4.0.5 [8]. For graphical data ggplot2 package version 3.3.3 was used [9].

### 2.3. Immunization Recommendations

There are no Europe-wide general vaccination recommendations for children. As a result, recommendations are made on a country-specific basis with many changes up to 2021. In Germany, the Standing Committee on Vaccination at the Robert Koch Institute is responsible, with annual recommendations. The Italian National Immunization Prevention Plan is released by the Ministry of Health and adopted by each region to its Regional Immunization schedule [10]. From 2014, the country-specific vaccination recommendations can be found on a website of the European Centre for Disease Prevention and Control [11]. Here is a simplified summarization with recommended age of vaccination for each country.

#### 2.3.1. Diphtheria, Tetanus, Pertussis, Poliomyelitis and Haemophilus Influenzae Type B

Vaccination against diphtheria (D), tetanus (T), pertussis (P), poliomyelitis (PV) and haemophilus influenzae type B (Hib), abbreviated with DTP-PV-Hib, is included in every national vaccine schedule, with adjustments in timing, antigen concentrations (e.g., diphtheria in child or adult dose) and addition of polio vaccination (attenuated or inactivated). Until 2013, vaccinations were given in France at 2, 3, 4 and 16 months. Since then, the vaccination has been discontinued at 3 months and the booster age has been reduced from 16 to 11 months. In Italy, vaccinations were given at 3, 5 and 11 months. In Lithuania, the basic immunization takes place at 2, 4, 6 and 18 months. In Germany, infants are vaccinated at 2, 3, 4 and 11 months, since 2020 the dose at 3 months is only recommended for premature infants.

#### 2.3.2. Hepatitis B

France, Lithuania and Italy recommend three ages for immunization: 2, 4 and 16 months. Since 2013, the age in France is 2, 4 and 11 months. Italian children are usually vaccinated at 3, 5 and 11 months. In Lithuania, vaccination with Hepatitis B starts after birth and is continued at ages 1 and 6 months. By contrast, in Germany the recommended ages are 2, 3, 4 and 11 months. As of June 2020, the second dose at 3 months is no longer required for full term infants.

#### 2.3.3. Pneumococcal Conjugate Vaccine

From 2005 to 2008, French infants usually received four doses of pneumococcal vaccine at the ages of 2, 3, 4 and 16 months. In 2009, this changed to 2, 3 and 12 months and was lowered from 12 to 11 months in 2013. Pneumococcal vaccination was included in the Italian immunization schedule in 2008, starting at the age of 3 months and followed by doses at 5 and 11 months. In Lithuania, infants are vaccinated at 2, 4 and 12 months. In Germany, since 2006, recommended ages are 2, 3, 4 and 11 months. However, since 2015, only preterm infants receive the dose at 3 months of age.

#### 2.3.4. Pneumococcal Polysaccharide Vaccine

Immunization with the 23-valent pneumococcal polysaccharide vaccine can start at 2 years of age, after completion of the pneumococcal conjugate vaccine. However, it is not included in the standard vaccination schedule of any countries in our analysis.

#### 2.3.5. Rotavirus Vaccine

Since February 2006, at least one rotavirus vaccine is licensed in the European Union. In Germany, the rotavirus vaccine was included in the standard vaccination schedule from 2013, starting at 6 weeks and continuing at 2 months of age. Italy introduced vaccination against Rotavirus in 2017 at 3 months of age. Since 2018 every child in Lithuania should be immunized at 2 months of age. For French children, rotavirus is not included in the vaccination schedule.

#### 2.3.6. Meningococcal Vaccine Serogroup C (MenC)

Since 2006, the meningococcal vaccine serogroup C, is recommended in Germany starting at 12 months of age. Italian children receive one dose at 13 months. France introduced the vaccination four years later, in 2017, and the age for the first dose was lowered to 5 months, with a second dose at 12 months. 

#### 2.3.7. Meningococcal Vaccine Serogroup B (MenB)

Since January 2013, a meningococcal vaccine against serogroup B has been available in the European Union. However, only Italy and Lithuania have included it in their standard vaccination schedule. In Italy, the vaccination was given from 2014–2016 at ages 7, 9 and 15 months, and since 2017, it has been implemented for ages 3, 4, 6 and 13 months. Lithuania has offered vaccination to children aged 3, 5 and 12 months since 2018.

#### 2.3.8. Meningococcal Vaccine Serogroup ACWY (MenACWY)

There are various vaccines available in the European Union for meningococcal vaccine against serogroup ACWY. In 2010 starting with Nimenrix has been offered since 2010 at 2 years of age and at 12 months of age since 2012. Four years later the initial age was lowered to at least 6 weeks. It is not included in the standard vaccination schedule in any country in our analysis.

#### 2.3.9. Measles, Mumps, and Rubella (MMR)

The vaccination against measles, mumps and rubella is available in all four countries in their regular vaccination schedule. Since 2001, immunization began in Germany at the age of 11 months and is completed at the earliest at 15 months. In France, vaccinations were given at 12 months and 3 years of age; the second dose has been administered at 16 months since 2005. Lithuanian children are vaccinated against MMR at 15 months and 6 years of age. Italy vaccinated at 13 months and 12 years of age until 2007, with the booster age reduced to 6 years from 2008.

#### 2.3.10. Varicella/Chickenpox Vaccine (VZV)

Immunization against chickenpox was introduced in Germany in 2004. A second dose after at least 4 weeks was recommended from 2006, if the first dose was combined with MMR. Since 2009, a refresher should always take place at the earliest at 15 months. In Italy, vaccinations against chickenpox are at the age of 13 months and 12 years, and since 2008, the booster has been given at the age of 6. There is no general vaccination recommendation against chickenpox in France and Lithuania.

#### 2.3.11. Human Papillomavirus (HPV) Vaccines

Vaccination against HPV was included in the general vaccination recommendations in France and Germany in 2007. French girls were first vaccinated when they were 14 years old, and since 2013 when they were 11 years old. In Germany, vaccination was initially recommended from 12 years of age, and from 2014 the minimum age was reduced to 9 years. Vaccination has also been recommended for boys since 2018. In Italy, the minimum age for vaccination is 11 years. As of 2017, both boys and girls aged 12 and over can be vaccinated against HPV. Lithuania introduced HPV vaccination only for girls in 2016 from the age of 11 years.

## 3. Results

### 3.1. Study Population

Vaccination records of 430 pediatric, liver transplant recipients, performed between January 2003 and April 2021, were available. The groups were divided depending on whether they had chronic (*n* = 363, 84.4%) or acute (*n* = 67, 15.6%) liver disease. More than 60% with chronic liver disease were diagnosed with biliary atresia (BA), followed by metabolic conditions with 9.6% and progressive familial intrahepatic cholestasis (PFIC) 6.9%. Further diagnoses were cryptogenic cirrhosis (6.3%), Alagille syndrome (3.6%), cystic fibrosis (3.3%) and other liver diseases (9.3%). Children with acute onset liver disease (*n* = 67) were diagnosed with hepatic malignancy (50.7%), acute liver failure (38.8%), neonatal onset (9%) and one patient with Amanita phalloides poisoning serving as a control group for further analysis. Distribution of gender did not significantly differ between both groups (49.3% male in chronic group, 58.2% male in control group; *p* = 0.181). Moreover, there were no significant differences in ages at time of transplantation. Baseline characteristics are presented in Table 1.

### 3.2. Analysis of Age-Appropriate Vaccination Coverage

Prior to transplantation, around 66.5% of children with chronic liver disease had received the recommended number of pneumococcal conjugate vaccine doses for their age, compared to 79.3% in the control group. This is similar with DTP-PV-Hib and Hepatitis B, although the overall vaccination rates are slightly higher compared to pneumococcal conjugate vaccine. Regarding the rotavirus vaccination, there is a significantly better vaccination coverage in the acute transplant recipients of 30.6% versus 16.6% in the chronic liver disease patients (*p* = 0.02). By contrast, significantly more children suffering from chronic liver disease (20.1%) were immunized with pneumococcal polysaccharide vaccine than in the control group (2.9%; *p* = 0.016). The same applies to Hepatitis A vaccination (42.0% versus 7.7%; *p* < 0.00001).

Around two thirds (65.7%) of eligible children with chronic liver disease were vaccinated against meningococci C, compared to 59.2% in the control group (*p* = 0.393). In meningococcal B, 22% of patients were vaccinated, compared to 16.1% in the control group, prior to transplantation after 2013 (*p* = 0.461). There is no significant difference between both groups regarding the quadrivalent meningococcal vaccine (ACWY), which has been available since 2010.

As a live vaccine, MMR post-transplant is not formally recommended, hence pre transplant vaccination is important: More than 81% of all children with chronic liver disease were up to date, compared to 62.3% of those with acute disease (*p* = 0.003). However, fewer children received age-appropriate vaccination against VZV, almost two thirds (65.2%) of children with chronic liver disease and 54.9% in the control group (*p* = 0.171).

The HPV vaccination was introduced quite late compared to the other vaccinations and is not carried out until at least the age of 9 years. Exactly 20% of all adolescents with chronic liver disease (*n* = 15) compared to none of the 2 possible patients received the HPV vaccination pre-transplant. The results are summarized in Table 2.

### 3.3. Country-Specific Vaccination Coverage between Healthy Children and Those with Chronic Liver Disease 

In order to minimize the country-specific influence on vaccination, and to check if parents are complying with early vaccine recommendations, the vaccination of eligible children with chronic liver disease was compared to healthy children (Table 3). Vaccination data on healthy children was taken from national reference databases as well as published data. Due to limited data, Lithuania was excluded from this comparison.

About 90% of healthy children in Germany born in 2016 [12], in France [13] and Italy [14] examined in 2018 received their first dose of MMR up to the age of 24 months. Similar rates are found for transplant candidates in Germany and France. Only in Italy is the rate lower at 60.4%. By contrast, rates of meningococcal vaccine serogroup C vaccination, which is usually administered at 12 months, are lower in transplant candidates in all three countries compared to healthy individuals at their second birthday [12,14,15].

While 83% received their second MMR dose by their second birthday in France, this is only true for half of eligible transplant candidates. Similarly in Italy, almost 60% of those with chronic liver disease and 90% of healthy controls were vaccinated twice with MMR by the age of seven. In Germany around 70% of healthy controls and transplant recipients had received MMR twice by their second birthday.

### 3.4. Age at Vaccination for 1st and 2nd Dose with MMR in Children with Chronic Liver Disease in Germany, France and Italy

**Figure 1:**
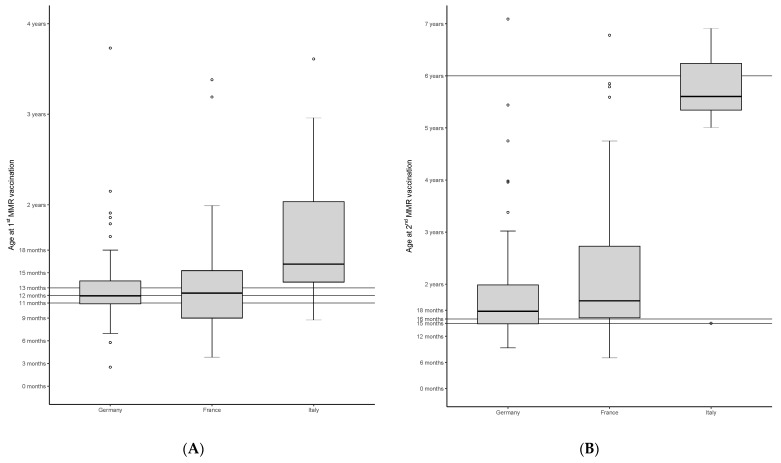
Boxplots with median age and interquartile range of vaccination age of 1st (**A**) and 2nd (**B**) MMR vaccines of children with chronic liver disease in Germany, France and Italy. Outliers are plotted as points. The horizontal lines mark the recommended age for vaccination: for first dose MMR in Germany 11 months, in France 12 months and in Italy 13 months, followed by a booster dose at 15 months, 16 months and 6 years respectively. The graph demonstrates the delayed/insufficient vaccination rate in these children in these three countries.

We analyzed the vaccination age of 1st and 2nd doses of MMR in children with chronic liver disease. As shown in Figure 1, median administration age for both vaccines is higher than the national recommendation. However, only median age of 2nd MMR dose in Italy is lower compared to the recommended age in the vaccination schedule.

### 3.5. Prevalence of Protective Antibody Levels against Hepatitis A and B, Measles and VZV Prior to Transplantation

In addition, antibody levels were measured prior to transplantation. All infants under 6 months of age were removed from the calculation in order to minimize the effects of the maternal loan titers. In the acute onset group, significantly more children had protective titers compared to children with chronic liver disease in Hepatitis B (80.0% and 63.3% respectively, *p* = 0.021). This is similar with VZV, where significantly fewer children and adolescents with chronic liver disease (53.1%) had sufficient pre-transplant titers compared to the acute onset group (68.8%, *p* = 0.042). By contrast, significantly more patients with chronic liver diseases had protective Hepatitis A antibodies compared to the acute onset group (60.1% versus 39.5%, *p* = 0.011). Comparison of the measles vaccination titers revealed no difference between both groups (62.9% in chronic, 70.7% in acute, *p* = 0.334). These results are summarized in Table 4.

### 3.6. Prevalence of Protective Antibody Levels in Infants with Age-Appropriate Vaccination

Interestingly, comparing the prevalence of protective vaccination titers only in those patients with age-appropriate vaccination revealed a difference in varicella zoster: significantly fewer children with chronic liver disease and age-appropriate vaccination with VZV had protective titers compared to the control group. Results are shown in Table 5.

## 4. Discussion

This study reviews the immunization status in 430 children and adolescents at the time of liver transplantation at five European liver transplant centers, revealing an under-immunization in this high-risk population. Only 80% of children with chronic liver disease were vaccinated against DTP-PV-Hib compared to national standards. However, this is in line with observations from Switzerland [7] as well as from the United States and Canada [6]. By contrast, levels of age-appropriate Hepatitis B-vaccinated children were 74.1% and lower compared to Feldman et al. with 84% of patients being age-appropriately vaccinated prior to transplantation. These results show that despite regular medical visits, vaccination recommendations are poorly implemented, even though national recommendations have been simplified and combination vaccines are available. This may also be a reflection of pediatrician concerns not to vaccinate due to liver disease. This is particularly worrying, as Leung et al. found insufficient antibody titers in in 67% of fully vaccinated children with Hepatitis B after liver transplantation [16]. Moreover, despite complete HBV vaccination, infection may occur post-transplant as case reports suggest [17]. 

A rotavirus infection is one of the leading infectious causes after pediatric solid organ transplantation [6]. However, rotavirus vaccination data is scarce in pediatric liver transplant patients. Since February 2006, a rotavirus vaccine has been authorized in Europe, however, it is not included in standard vaccination schedules in every country. The number of vaccinated patients (30.6%) in our control group is significantly higher compared to patients with chronic liver disease (16.6%; *p* = 0.02). The lesser number of children vaccinated may be a reflection of the fact that diagnosis of chronic liver disease is often made in the first few weeks of life due to jaundice [18] and, consequently, within the narrow timeframe of the rotavirus vaccination. Interestingly, only 59% of hepatologists from the SPLIT group recommended rotavirus vaccine for infants prior to transplantation [19]. The situation is similar with pneumococcal conjugate vaccine, in which only two thirds of children with chronic liver disease were up to date pre-transplantation. From the second birthday, vaccination status can be extended with the 23-valent pneumococcal polysaccharide vaccine. As an indication vaccination, it also explains why almost 20% of all chronic liver disease patients are significantly better vaccinated than in the control group (2.9%, *p* = 0.02). Data in adults with liver cirrhosis suggests that they have lower antibody levels to pneumococcal capsular polysaccharide after vaccination, compared to healthy individuals, and that after transplantation these drop to pre-vaccination levels [20].

An infection with chickenpox can have a prolonged, severe course under immunosuppression [21,22] and is one of the leading causes of VPI following pediatric solid organ transplantation [5]. Live-attenuated vaccines are not generally recommended in immunosuppressed patients, and vaccination with MMR and varicella should not be given until the age of 6 months at the earliest [23]. However, the window of opportunity for vaccination is usually limited prior to transplantation because pediatric liver transplant candidates often have unstable disease courses. Thus, it is essential to immunize eligible children at an early age. Our data shows that children suffering from chronic liver disease showed a median vaccination age with MMR, which is close to the national recommendations (Figure 1). Moreover, vaccination rates were better compared to the control group: 81% versus 62% (*p* = 0.003) for MMR and 65% versus 54.9% (*p* = 0.171) for varicella were up to date pre-transplant. However, this is less than the 90% of all patients vaccinated on time with both live vaccines prior to transplant observed by Feldman et al. [6].

On the one hand, the median age of the vaccinated children is close to the national recommendations; on the other, the country-specific comparison of vaccinations shows that this is far below the national reference data for Italy, but also for the second MMR dose in France. This may be a country-specific reflection of vaccination hesitancy, which recently led to mandatory vaccinations in France [24], Italy [25] and Germany [26]. In addition, there is a wide variation in immunization practices for pediatric liver transplant candidates. For example, 15% of pediatric transplant hepatologists always recommend live vaccines and only 84% sometimes [19]. In view of this data, the information that these live vaccinations should be carried out quickly does not seem to reach all families, even if the coverage with MenC is still lower in all countries (Table 3). To improve this, several factors must be addressed: vaccination education of parents [27] and co-treating pediatricians, reminder systems for upcoming or missed vaccination windows [28], digitally available vaccination records as well as standardization of immunization schedules.

Vaccination titers can be used for a better assessment of vaccination status. The group with chronic liver disease had significantly better titers for Hepatitis A, but also had significantly better coverage for indication vaccines. Interestingly, the prevalence of seroprotective titers for Hepatitis B and VZV is higher in children with acute liver disease than in children with a chronic disease (Table 4), but with no difference for measles. However, this may also represent a poorer response to vaccinations in the context of liver disease, as children with biliary atresia show diminished humoral immunity to MMR and VZV vaccines compared to healthy controls [29]. If only those children who were vaccinated according to their age are examined, significantly fewer children with chronic liver disease had protective titers compared to the control group (Table 5). However, after transplantation, titers may fall due to immunosuppression. In a Swiss study, only children with a history of chickenpox had detectable VZV-antibodies and those previously vaccinated did not [7]. Yoeli et al. demonstrated that non-immune VZV-patients, after liver transplantation, received less doses prior to transplantation, were younger at transplantation and had less time between their last VZV-dose and transplantation [30]. Thus, this represents a balancing act, that vaccination should take place early enough, but not too early, so as not to compromise the success of vaccination. Therefore, regular vaccination titers can be measured in order to document response on the one hand, and on the other hand, the need for vaccine refreshment before transplantation, as described by L Huillier et al. [7]. However, cellular-mediated aspects of immunity are disregarded by only measuring antibody titers [31] and even patients with non-protective antibody titers may mount an effective immune reaction.

The current study has some limitations: As this is a multicenter investigation, it has the problem that national vaccination calendars are different. This means that vaccination priorities and times in each country have shifted several times over the study period of more than 18 years. For example, rotavirus vaccine is not included in all countries analyzed and an indication vaccination does not necessarily have to be taken over by the health system, so that this may be omitted by the families for financial reasons. In addition, the willingness to vaccinate varies; on the one hand, from country to country and, on the other hand, over time, resulting in recent mandatory vaccinations in France and Italy [24,25]. In Germany, the measles vaccination became mandatory for entry into kindergarten or school in March 2020 [26]. In addition, the total level of vaccination titers were not investigated in this study, as the comparability is limited, due to the different laboratories and measurement methods over time. Those patients with acute liver disease were defined as a control group in this study, but their vaccination titers may also be subject to changes due to the underlying disease. Moreover, the level of the vaccination titer can differ significantly, even if the number of patients with a protective level does not differ. 

In conclusion, incomplete vaccination status and insufficient antibody levels are common prior to pediatric liver transplantation. As infection is relevant for morbidity and mortality in the short and long term after transplantation, new strategies should be found, in particular, to reduce VPI. Mandatory vaccination may be a start, but improvements in the vaccination education of parents and pediatricians to enhance acceptance, as well as reminder systems of vaccination windows seem useful. Moreover, standardized vaccination recommendations across Europe, including new vaccines (e.g., rotavirus), with digitally accessible vaccination records as well as regular serological analyses of vaccination titers prior to transplantation may be helpful here, with re-vaccination if necessary.

## Figures and Tables

**Table 1:** Patient data of 430 children who underwent liver transplantation between January 2003 and April 2021 from five centers in Europe.

	Chronic Liver Disease (*n* = 363)	Control Group/Acute Liver Disease (*n* = 67)	*p*
Gender, male (%)	179 (49.3%)	39 (58.2%)	0.181
Diagnosis	BA: 221 (60.9%) Metabolic: 35 (9.6%) PFIC: 25 (6.9%) Cryptogenic cirrhosis: 23 (6.3%) Alagille: 13 (3.6%) CF: 12 (3.3%) Other: 34 (9.3%)	Malignancy: 34 (50.7%) Acute liver failure: 26 (38.8%) Neonatal onset: 6 (9.0%) Amanita phalloides poisoning: 1 (1.5%)	
Country distribution of patients	Germany: 163 (44.9%) France: 106 (29.2%) Italy: 84 (23.1%) Lithuania: 10 (2.8%)	Germany: 42 (62.7%) France: 8 (11.9%) Italy: 16 (23.9%) Lithuania: 1 (1.5%)	
Age at time of transplant, median (IQR)	1.6 (0.7–4.8)	2.2 (1.3–4.7)	0.314
Year of birth, median (IQR)	2011 (2007–2015)	2011 (2007–2015)	0.849

**Table 2:** Age-appropriate vaccination in children with chronic and acute liver disease. Data expressed as percentage of age-appropriate vaccination (number of age-appropriate vaccinated children/total number of eligible children).

Vaccine	Chronic Liver Disease	Control Group	*p*
DTP-PV-Hib	80.2% (291/363)	85.5% (53/62)	0.324
Hepatitis B	74.1% (269/363)	79.0% (49/62)	0.409
Hepatitis A	42.0% (100/238)	7.7% (4/52)	<0.00001
Pneumococcal conjugate vaccine	66.5% (230/346)	79.3% (46/58)	0.052
Pneumococcal polysaccharide vaccine	20.1% (30/149)	2.9% (1/34)	0.016
Rotavirus (since 2006)	16.6% (46/277)	30.6% (15/49)	0.020
MenC	65.7% (134/204)	59.2% (29/49)	0.393
MenB (since 2013)	22.0% (40/182)	16.1% (5/31)	0.461
MenACWY (since 2010)	8.1% (19/236)	6.7% (3/45)	0.751
HPV	20.0% (3/15)	0.0% (0/2)	Ø
MMR	81.1% (198/244)	62.3% (33/53)	0.003
VZV	65.2% (144/221)	54.9% (28/51)	0.171

**Table 3:** Country-specific vaccination of eligible children with chronic liver disease and healthy controls. Data on healthy children was taken from national reference databases.

	Germany		France		Italy	
	Chronic Transplant Candidates	Healthy Children [12]	Chronic Transplant Candidates	Healthy Children	Chronic Transplant Candidates	Healthy Children [14]
MenC received in % up to 24 months of age	44.8%	77.8%	40.5%	65.3% [15]	43.6%	87.8%
1st MMR received in % up to 24 months of age	86.5%	89.8%	90.2%	90.9% [13]	60.4%	94.1%
2nd MMR received in % up to 24 months of age	65.8%	69.9%	50.7%	83.4% [13]	57.9% up to 84 months	90.1% up to 84 months
1st VZV received in % up to 24 months of age	81.1%	83.7%	46.7%	%	46.8%	46.7%
2nd VZV received in % up to 24 months of age	46.2%	66.0%	10.5%	%	26.3% up to 72 months	%

**Table 4:** Prevalence of seropositive rates of IgG antibodies before transplantation in infants aged 6 months and older. Data is expressed as percentage of protective titers (number of age-appropriate vaccinated children/total number of investigated children).

Vaccine	Chronic Liver Disease	Control Group	*p*
Hepatitis B	63.3% (195/308)	80.0% (40/50)	0.021
Hepatitis A	60.1% (169/281)	39.5% (17/43)	0.011
Measles	62.9% (163/259)	70.7% (29/41)	0.334
VZV	53.1% (163/307)	68.8% (33/48)	0.042

**Table 5:** Prevalence of seropositive rates of IgG antibodies before transplantation in age-appropriate vaccinated infants. Data is expressed as percentage of protective titers (number of age-appropriate vaccinated children with protective titer/total number of investigated children with available titer prior to transplantation).

Vaccine	Chronic Liver Disease	Control Group	*p*
Hepatitis B	68.6% (131/191)	81.1% (30/37)	0.127
Hepatitis A	88.9% (80/90)	100.0% (4/4)	1.00
Measles	80.6% (125/155)	72.7% (16/22)	0.388
VZV	67.4% (91/135)	80.8% (21/26)	0.006

## Data Availability

All data requests should be submitted to the corresponding author for consideration. Access to anonymized data may be granted, following review.
